# Postural Control during the Stroop Test in Dyslexic and Non Dyslexic Teenagers

**DOI:** 10.1371/journal.pone.0019272

**Published:** 2011-04-27

**Authors:** Zoï Kapoula, Eric Matheron, Emilie Demule, Caroline Fauvel, Maria-Pia Bucci

**Affiliations:** 1 IRIS Laboratory CNRS/FRE 3375, Université Paris VII – Assistance Publique Hôpitaux de Paris, Paris, France; 2 University of Paris V, 12, rue de l'Ecole de médecine, Paris, France; 3 Ecole des psychologues praticiens, Université Catholique de Paris, Paris, France; University of Rennes 1, France

## Abstract

Postural control in quiet stance although simple still requires some cognitive resources; dual cognitive tasks influence further postural control. The present study examines whether or not dyslexic teenagers experience postural instability when performing a Stroop dual task for which their performances are known to be poor. Fifteen dyslexics and twelve non-dyslexics (14 to 17 years old) were recruited from the same school. They were asked to perform three tasks: (1) fixate a target, (2) perform an interference Stroop test (naming the colour or the word rather than reading the word), (3) performing flexibility Stroop task: the subject performed the interference task as in (2) except when the word was in a box, in which case he had to read the word. Postural performances were measured with a force platform. The results showed a main task effect on the variance of speed of body sway only: such variance was higher in the flexibility task than for the other two tasks. No group effect was found for any of the parameters of posture (surface, mediolateral and anteroposterior sway, variance of speed). Further wavelet analysis in the time-frequency domain revealed an increase in the spectral power of the medium frequency range believed to be related to cerebellum control; an accompanying increase in the cancellation time of the high frequency band related to reflexive loops occurred for non-dyslexics only. These effects occurred for the flexibility task and could be due to its high cognitive difficulty. Dyslexics displayed shorter cancellation time for the medium frequency band for all tasks, suggesting less efficient cerebellar control, perhaps of eye fixation and attention influencing body sway. We conclude that there is no evidence for a primary posture deficit in 15 year old teenagers who come from the general population and who were recruited in schools.

## Introduction

Postural control in quiet stance involves continuous multisensory central integration of visual, vestibular and proprioceptive inputs in order to produce motor commands controlling the body's position in space. Although it is a simple task, body control still requires some cognitive resources. In everyday life cognitive and attentional resources needed to control posture are usually divided in order to perform other tasks simultaneously (conversation, listening, thinking etc.). Thus postural control in quiet stance is naturally part of dual or multiple tasks. The question arises whether under such ecologic conditions postural control in quite stance is impaired. Many studies used double tasks contributing greatly to the field of postural control [1], [2]. It has been showed, in adults or elderly, that the cognitive task influences postural control [2], [3], [4], [5], [6]. Interestingly, cognitive tasks can either deteriorate or improve posture stability [4], [5], [6]. Various models have been proposed to explain such interaction: for instance the model of competition or sharing the attention resource system, the model of non-linear interaction between different tasks and the model of priority task [7], [8].

In the present study we examine postural control in quiet stance in dyslexic and non dyslexic teenagers while performing the Stroop test which is itself a double task as it will be explained below. The Stroop test was introduced by J.R. Stroop in 1935. It is widely used as it allows examination of the interference between two tasks. The subject must inhibit an automatic response (reading) and give less obvious response (color denomination). This test allows the evaluation of deficits of selective attention, which is the capacity to maintain attention to a given target in the presence of distraction, or to take into account one dimension of the stimulus, ignoring the other dimension. Olivier et al. [8] examined postural control in eight children five to nine years old using a double task with a modified Stroop test. They reported deterioration of postural stability of the children while doing the Stroop test; deterioration increased when the posturography was done with vibration of the feet. In adults, in contrast, no deterioration was observed.

Our use of the Stroop test as a double task is motivated by the fact that dyslexics are known to have difficulty with such test. Indeed, Protopapas et al. [9] compared dyslexics and non-dyslexics (mean age 12.5 years), as well as children from the general school population according to their reading skills. They reported greater interference in the Stroop test in teenagers with dyslexia, as well as in poor readers. Faccioli et al. [10] also reported more interference in dyslexic children (7–11 years old). Finally, Kapoula et al. [11] reported that greater interference in the Stroop test persists even for older dyslexics (15 years old). All these observations are against earlier opposite expectations [12] for less interference in the Stroop test for dyslexics due to their reduced reading automaticity. As suggested by Protopapas et al. [9] the Stroop test and reading share common executive functions, mainly attention and inhibitory control and this would explain why poor readers show more interference errors in the Stroop test. Another controversial issue is that of reduced mental flexibility in dyslexia. Stoet & Snyder [13] reported that dyslexics may have impaired capacity to rapidly shift their visual attention in a task-switching paradigm; yet, the deficit, according to these authors, would be more at the peripheral neural pathways such as the magnocellular layers in the lateral geniculate nucleus rather than at a central cognitive level. This idea contrasts earlier reports from Helland and Asbjornsen [14], Brosnan et al. [15] suggesting a problem in shifting attention at a higher level. In our prior study of Stroop performance in dyslexia [11] we used a version of the test with four cards: color naming, reading, interference and flexibility. In the latter, teenagers had to name the color of the word inhibiting reading except when the word was inside a box. This flexibility task enables the testing of cognitive switching between tasks. It was found that dyslexics did not have more difficulty with this task than with the interference task, arguing against specific problems with mental switching.

Let us now return to a brief review of studies on postural control in dyslexia. Kapoula and Bucci [16] measured postural control in younger dyslexic teenagers (average age 13 years old) while fixating at two distances, 200 cm and 40 cm with eyes open and eyes closed. Dyslexics were more unstable during such fixation tasks whatever the distance, far or near. Nevertheless, when they were asked to make active vergence eye movements between a near and a far target (convergence-divergence), their postural stability improved and became almost normal, while no significant change was observed for the control teenagers. Moreover, a separate eye movement study with videoculography showed marked fixation instability for dyslexics in the simple task requiring prolonged fixation similarly to single posturography testing conditions. Thus, Kapoula and Bucci [16] concluded that rather than a primary postural syndrome, dyslexic teenagers have unstable ocular fixation, particularly reduced capacity to maintain the angle of vergence at the required depth, and this might influence their postural stability. Unstable fixation may be due to attention fluctuation. Performing actively vergence eye movements engages visual attention thus leading to better posture stability. This interpretation contrasts other studies suggesting postural deficiency in dyslexics. For instance, Quercia et al. [17], Pozzo et al. [18] suggested that there is a postural deficiency syndrome in dyslexia that is an alteration of postural equilibrium accompanied by deficit of ocular capabilities due to a defect of proprioceptive and visual information.

Other studies examined motor balance. Bear in mind however, that although postural control in quiet stance and balance control tasks are related the sensorimotor mechanisms involved are not identical. Stoodley et al. [19] examined balancing abilities (standing either on the right or the left foot) and recorded body motion. It was found that with their eyes open, dyslexic children (mean age 10.8 years) were significantly less stable than control children. Although not all dyslexic children exhibited such balance impairment a correlation between reading performance, spelling errors and balancing abilities with eyes open was found. The authors attributed impaired balancing to cerebellum deficiency and magnocellular immaturity. Earlier, Nicolson & Fawcett [20] examined balance control in 13-year-old dyslexics and non dyslexics using many double tasks (counting backward, auditory choice reaction, while either on one foot, or with both feet); they used video recording of the performance and evaluated the clumsiness index. Under most of the dual tasks balance was significantly impaired for the dyslexic group, while controls showed no such impairment. The authors suggested that dyslexics need to invest conscious resources in order to monitor balance, and thus their performance is affected by the secondary task which distracts attention away from the balance task.

To summarize, the existing studies on dyslexia, use different age groups, different methods (posture, balance, different measures) rendering it difficult to compare them. Nevertheless some problems, which present themselves do appear to be task specific. The specific question here concerns whether or not 15-year-old dyslexic teenagers experience postural control deficit during a dual task like the Stroop which is highly cognitively demanding and for which dyslexics are known to perform poorly. Dyslexics without hyperactivity nor dyspraxia were recruited from the same school as non-dyslexics. We studied this question with measures and analysis of standard posture parameters (surface, lateral and anterior/posterior oscillations of the centre of body pressure, and variance of speed). In addition to these basic parameters we also applied a wavelet analysis to assess frequency of body sway in the time domain. A first prediction would be that dyslexics would exhibit postural instability as the Stroop test is more difficult for them. Yet, based on our prior study with 13-year-old dyslexics [16] we predict that involvement of dyslexics in the high demanding Stroop tasks would lead to task dependent but normal postural stability. The idea being, that as long as dyslexics are actively involved in a task, mobilizing their attention and their cognitive resources, their postural control should be normal irrespective of their performance in the Stroop test. In other words, as in our prior study with younger teenagers, we expected the cognitive dual tasks to reduce differences in postural performances between dyslexics and non dyslexics. The results show no difference between the two groups, with the exception of some subtle differences revealed by the wavelet analysis in the frequency-time domain.

## Methods

### Ethics Statement

The postural control investigation complied to the tenets of the Declaration of Helsinki and was approved by the local human experimentation committee, the “Comité de Protection des Personnes” (CPP) Ile de France VI (No: 07035), Necker Hospital in Paris. Written informed consent was obtained from children's parents after the nature of the procedure had been explained.

### Subjects

Twenty-seven young adolescent subjects (6 females, 21 males) in the age range of 14–17 years were recruited all from the college of St Sulpice in Paris. Fifteen were subjects with Dyslexia (3 females, 12 males) in the age range of 14–17 years (15.6±0.9 years), and 12 control subjects (3 females, 9 males) in the age range of 14–17 years (15.0±1.0 years); girls were a minority both in the dyslexic (25%) and non-dyslexic group (33%).

All teenagers were perfectly able to see the targets used and to read the words for the Stroop tests. Dyslexics were admitted to this college because of known dyslexia. They underwent extensive examination, including neurological/psychological and phonological tests, conducted in the current year of the present study; for each teenager, their speed of reading, text comprehension, as well as their capacity to read word/pseudo words was evaluated by using the L2MA battery [21]. This is the standard test developed by the applied psychology centre of Paris, and is used extensively in France. Inclusion criteria were: scores in these tests beyond two standard deviations; a normal mean intelligence quotient (IQ, evaluated with WISC III), i.e. between 85 and 115. Attention and concentration problems absent any signs of hyperactivity were present in 6 of the dyslexics (3 with severe dyslexia and 3 with moderate); no teenager had dyspraxia. At the college, they followed the same educational program as the other pupils with the exception of additional classes for improving reading and orthography skills; in parallel they followed individual training with orthophonist. Difficulties in reading were still present, severe for 5 of them; problems with orthography were present for 4 of the dyslexics

Non-dyslexic teenagers had to satisfy the following criteria: no known neurological or psychiatric abnormalities, no history of reading difficulty, no visual stress or any difficulties with near vision. IQ and reading measurement could not be applied for these teenagers. It should be noted that there is no evidence for a correlation between intelligence and Stroop performances [9]. Neither there is evidence that posture control depends on intelligence. Non-dyslexics were selected by the director of their school on the basis of their school performances; their score in French (reading, understanding, and orthography), mathematics and foreign languages were all above the mean score of the class; their reading score was higher than that of dyslexics. Recruitment for non-dyslexic teenagers, based on school performance alone has been used by others [22], [23], [24]. Reading scores were higher for controls than for dyslexics.

### Platform characteristics

To measure postural stability, we used a force platform (principle of strain gauge) consisting of two dynamometric clogs (Standards by Association Française de Posturologie; produced by TechnoConcept, Céreste, France). Body sway was evaluated by computing the excursions of the center of pressure (CoP) measured over a period of 25.6 s; the equipment contained an Analog–Digital converter of 16 bits and the sampling frequency of the CoP was 40 Hz.

### Visual target and Stroop tests

**Figure 1:**
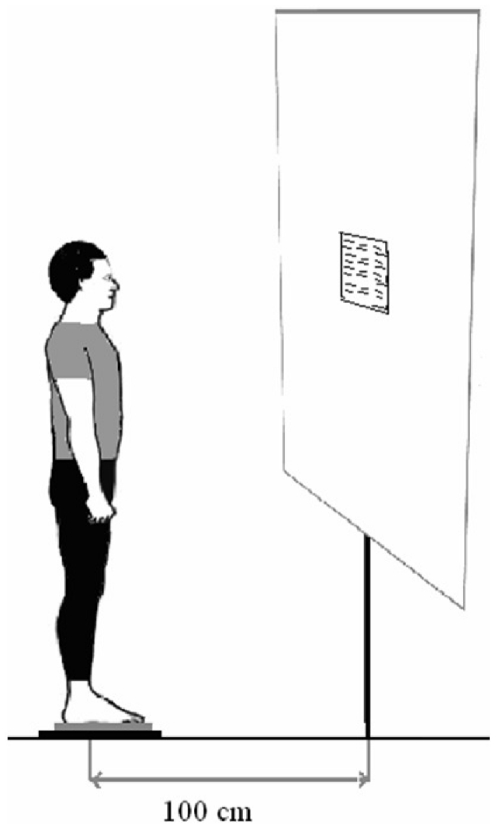
Illustration of posturography testing conditions. The subject viewed the Stroop test on the screen at 100 cm, at the eye level.

The visual target or the Stroop card was placed at eye level for each subject standing upright on the force platform (see Figure 1). The visual target was a cross “x” for the fixating control task. For the Stroop task we used a series of 40 words displayed over 10 lines; two cards were used, one with the words written in different colors (interference task), the other with some of the words being in boxes (flexibility task).

For the “interference task” teenagers had to name loudly the color of the print of the words, printed in an incongruent color (red, green, blue or yellow). For instance, blue printed in red ink. The “flexibility task” was similar to the last except that the teenagers had to read the word rather than name its color when the word was inside a box. The errors made on interference and flexibility conditions were recorded.

### Testing conditions

Upright stance posturography was carried out with subjects placed on the force platform; they looked at the cross target or at Stroop cards placed at one meter at eye level (Figure 1); posturography was done for a duration of 25.6 seconds for each condition. Such short duration was used to avoid problems with sustained attention particularly in the cross fixation task. The order of the 3 conditions was the following: first the fixation task (FT), second, the Stroop interference task (SIT), and third, the Stroop flexibility task (SFT). A one-minute rest period was applied between any two conditions: the subjects sat on a chair.

### Postural parameters

We analyzed the surface of the CoP excursions, the standard deviations of lateral (SDx) and anteroposterior (SDy) body sways and the variance of speed. The surface area was measured with the confidence ellipse including 90% of the CoP positions sampled eliminating the extreme points [25].

### Frequency analysis

We applied a wavelet non linear analysis to study frequency in the time domain. Applied to CoP displacements, the wavelet transform elaborates a time-frequency chart of body sway [26], [27]. The wavelet analysis used by the software is a continuous one. The mother wavelet used is Morlet. The time-frequency plane's principle advantage is its double resolution (time and frequency). Thus, the fact that the spectrum of the body sway is not constant over the test time is proved. The wavelet analysis was applied on the anteroposterior and mediolateral sway data. The spectral power was calculated for the frequency bands 0.05–0.5 Hz (F1), 0.5–1.5 Hz (F2), higher than 1.5 Hz (F3) on the anteroposterior and mediolateral sways as power indices (PIy and PIx, respectively). The hypothetical physiological significance of the spectral power of different bands is the same as for the FFT i.e. 0–0.5 Hz visual-vestibular [28], [29], [30], 0.5–1.5 Hz cerebellar [30], >1.5 Hz reflexive loops [7], [27]. As a rule, power in the higher band (F3) is minimal in healthy subjects during quiet standing, but it can be observed with aging, and postural pathology, or in dynamic postural conditions [27].

Moreover, the canceling time (CT) of each frequency band was also calculated for the anteroposterior (CTy) and mediolateral (CTx) sway, i.e. the total time during which the spectral power of the body sway for the frequency range was cancelled by the posture control mechanisms; the longer the canceling time of a frequency band, the better the posture control [26], [27]. In general, only a few frequencies are cancelled and not the complete band. The fact that a certain frequency has its power reduced to zero over a period of time shows that there has been a successful action of the postural control system since the overall entropy of the sway is reduced (this implies that there is an external action - which is the control system's action). While most healthy subjects exhibit these zero power instances in their postural sway spectrum, the pathological subjects cannot. It still remains to be proven how the cancelled frequencies are “chosen” by the postural control system, but it may be assumed that the choice criterion is the minimization of the muscular effort for controlling the sway.

The postural instability index (PII) quantifying the postural performance, taking into account the two precedent indices (PI and CT), was also calculated [26], [27] as the following:

PII = Σ_x_Σ_y_PI(F1, F2, F3)/CT(F1, F2, F3).

In healthy adults and during the single quiet stance task the PII is close to unity (see [27]. This additional analysis and associated parameters were obtained with the software PosturoPro (Framiral, Cannes, France, www.framiral.fr).

### Statistical analysis

Statistical analysis was performed using the GLM (General Linear Models, procedure of SAS/STAT, release 9.1). Parameters describing postural control during quiet standing were analyzed using repeated-measures analysis of variance (ANOVA – type III error) with group (dyslexics and controls) as the inter-subject factor, and with the task as within subject factor (fixation task – FT, the Stroop interference task – SIT) and the Stroop flexibility task – SFT). Because only two or three data groups were compared, post hoc comparisons were done whenever necessary using the Fisher's PLSD test, with P<0.05 considered as significant. Stroop performances were not included in the analysis as the test was not complete (i.e., only the interference and flexibility tasks were included and with 40 words only). The focus of the study is on posture control during such task rather than evaluating Stroop performances per se as this has been done in other studies specifically designed [11].

## Results

### Postural results

Means and standard errors are shown in Table 1: for each group of subjects (Dyslexics and Controls) and for the 27 subjects together, and for each condition (FT, SIT, SFT); all postural parameters are shown, i.e. the surface of the CoP excursions, SDx, SDy, the variance of speed, PII, and PI and CT for each plane (respectively PIy, PIx, CTy and CTx) for each frequency bands (0.05–0.50 Hz, 0.50–1.50 Hz and 1.50–10.00 Hz).

**Table 1:** Postural stability measurements in upright stance (25.6 s. duration).

		Controls	Dyslexics	Total
**Surface (mm^2^)**	*Fixation Task*	176.85±21.73	190.87±34.04	184.64±20.91
	*Interference*	166.80±27.47	258.49±81.64	217.74±47.07
	*Flexibility*	346.92±98.37	286.83±83.30	313.54±62.69
**SDy (mm)**	*Fixation Task*	4.30±0.36	4.64±0.53	4.49±0.33
	*Interference*	3.71±0.25	4.93±0.99	4.39±0.56
	*Flexibility*	6.38±1.53	5.15±1.10	5.70±0.90
**SDx (mm)**	*Fixation Task*	3.19±0.33	2.93±0.24	3.04±0.20
	*Interference*	3.18±0.43	3.24±0.38	3.22±0.28
	*Flexibility*	3.71±0.42	3.63±0.45	3.67±0.31
**Speed Variance (mm^2^/s^2^)**	*Fixation Task*	73.74±14.14	69.62±20.42	71.45±12.75
	*Interference*	102.64±24.04	119.07±43.87	111.77±26.21
	*Flexibility*	234.90±79.54	182.25±72.98	205.65±53.00
**Wavelets PII**	*Fixation Task*	1.60±0.11	1.64±0.17	1.61±0.10
	*Interference*	1.71±0.13	1.68±0.19	1.69±0.12
	*Flexibility*	2.17±0.22	1.90±0.21	2.02±0.15
PIy (mm^2^*10^6^) 0–0.5 Hz	*Fixation Task*	66.03±1.93	66.32±1.68	66.19±1.24
	*Interference*	64.78±2.54	65.82±2.36	65.35±1.70
	*Flexibility*	68.31±2.13	67.88±2.03	68.07±1.45
**0.5–1.5 Hz**	*Fixation Task*	55.94±1.73	54.72±1.73	55.26±1.21
	*Interference*	56.22±2.18	56.25±2.14	56.24±1.50
	*Flexibility*	59.21±2.05	57.73±1.86	58.50±1.36
**>1.5 Hz**	*Fixation Task*	39.65±2.06	37.93±1.86	38.69±1.36
	*Interference*	40.29±2.54	39.26±2.38	39.72±1.71
	*Flexibility*	42.06±2.37	40.72±1.71	41.32±1.39
PIx (mm^2^*10^6^) 0–0.5 Hz	*Fixation Task*	69.96±1.51	70.68±2.03	70.36±1.29
	*Interference*	69.81±1.29	72.29±2.11	71.19±1.30
	*Flexibility*	75.75±3.19	74.80±2.65	75.22±2.01
**0.5–1.5 Hz**	*Fixation Task*	60.04±0.99	59.29±1.87	59.62±1.11
	*Interference*	60.01±1.12	61.24±1.89	60.69±1.15
	*Flexibility*	65.36±2.46	64.27±2.46	64.76±1.72
**>1.5 Hz**	*Fixation Task*	43.19±0.71	43.30±2.20	43.25±1.24
	*Interference*	44.27±1.15	44.81±1.98	44.57±1.19
	*Flexibility*	49.71±2.52	46.27±2.29	47.80±1.70
**Wavelets CTy (s) 0–0.5 Hz**	*Fixation Task*	0.73±0.14	0.74±0.13	0.74±0.10
	*Interference*	0.92±0.21	1.12±0.38	1.03±0.23
	*Flexibility*	0.67±0.23	0.87±0.19	0.78±0.14
**0.5–1.5 Hz**	*Fixation Task*	0.79±0.14	0.74±0.11	0.76±0.09
	*Interference*	1.19±0.18	0.89±1.14	1.02±0.11
	*Flexibility*	0.95±0.19	0.99±0.14	0.97±0.12
**>1.5 Hz**	*Fixation Task*	0.02±0.01	0.01±0.00	0.01±0.00
	*Interference*	0.02±0.01	0.02±0.01	0.02±0.01
	*Flexibility*	0.02±0.01	0.02±0.01	0.02±0.01
**CTx (s) 0–0.5 Hz**	*Fixation Task*	0.49±0.12	0.59±0.12	0.54±0.09
	*Interference*	0.56±0.11	0.43±0.09	0.49±0.07
	*Flexibility*	0.27±0.07	0.45±0.10	0.37±0.06
**0.5–1.5 Hz**	*Fixation Task*	1.62±0.31	1.05±0.12	1.30±0.16
	*Interference*	1.58±0.22	1.28±0.20	1.41±0.15
	*Flexibility*	1.50±0.27	1.02±0.16	1.23±0.15
**>1.5 Hz**	*Fixation Task*	0.01±0.01	0.02±0.01	0.02±0.01
	*Interference*	0.02±0.01	0.02±0.01	0.02±0.00
	*Flexibility*	0.07±0.03	0.01±0.01	0.04±0.01

For 15 and 12 control adolescents and for the 27 subjects together. Means and standard errors of surface of CoP, standard deviations of lateral (SDx) and of anteroposterior (SDy) body sway, variance of speed, PII, and PI and CI for each plane (respectively PIy, PIx, CIy and CIx) for each frequency bands (0.05–0.50 Hz, 0.50–1.50 Hz and 1.50–10.00 Hz) for each condition i.e. the quiet fixation task (FT), the Stroop interference test (SIT) and the Stroop flexibility test (SFT).

P-values obtained when ANOVA was performed on each postural parameter with the group and task factors are all shown in Table 2. The following significant effects were found.

**Table 2:** P-values obtained when ANOVA was performed.

	p Group	p Task	p Task*Group
**Surface mm^2^**	0.783	0.123	0.533
**Sdy (mm)**	0.881	0.231	0.418
**Sdx (mm)**	0.765	0.257	0.914
**Speed Variance (mm^2^/s)**	0.745	0.024*	0.782
**Wavelets PII**	0.559	0.055°	0.660
**Ply 1**	0.865	0.411	0.943
**Ply 2**	0.611	0.244	0.932
**Ply 3**	0.443	0.483	0.988
**Plx1**	0.686	0.068	0.750
**Plx2**	0.900	0.025*	0.815
**Plx3**	0.572	0.056°	0.555
**CTy1**	0.327	0.413	0.880
**CTy2**	0.411	0.136	0.477
**CTy3**	0.120	0.698	0.646
**CTx1**	0.559	0.211	0.281
**CTx2**	0.012*	0.731	0.826
**CTx3**	0.045*	0.032*	0.009*

On the studied postural parameters for group (dyslexics vs. controls), task (quiet fixation task, Stroop interference test and Stroop flexibility test) and group-task interaction effects. Asterisk indicates significant difference (p<0.05) and circle indicates marginally significant effects.

### Effects of task

**Figure 2:**
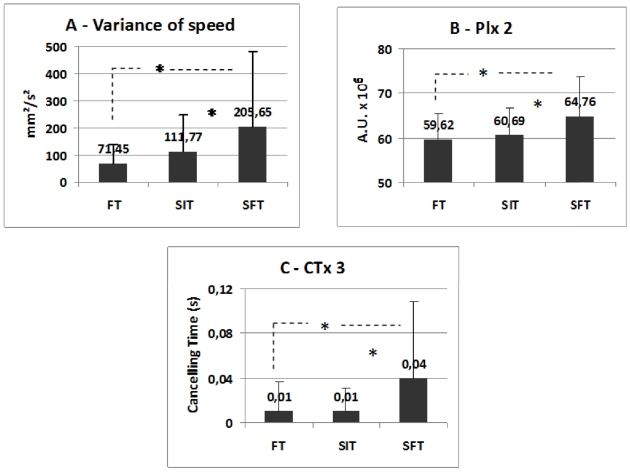
Effects of task. The fixation task (FT), the Stroop interference test (SIT) and the Stroop flexibility test (SFT) in all teenagers on the variance of speed (**A**), and on two parameters elaborated from the wavelet transform, the power indices for the second frequency band (**B** - PIx2) and the canceling time of the third frequency band (**C** - CTx3) on the mediolateral sway during upright stance posturography (**B** and **C**, respectively).

There was a main effect of the task only on the variance of speed (F_(2,50)_ = 3.90; p = .024), and on two parameters elaborated from the wavelet transform, the power indices for the second frequency band (PIx2, F_(2,50)_  = 3.90; p = .025) and the canceling time of the third frequency band (CTx3, F_(2,50)_  = 3.62; p = .032) on the mediolateral sway. The Fisher's PLSD post hoc showed significant increment of the variance of speed and PIx2 for the SFT (p<0.05) compared to the FT and with the SIT (see Fig. 2a,b). The CTx3 was longer for the SFT (p<0.05) than for the other tasks (see Fig. 2c).

Table 2 also shows a marginally significant effect for the PII (F_(2,50)_  = 3.01; p = .055), and PIx3 concerning the high frequency band (F_(2,50)_  = 2.99, p = .056); these parameters trended to be higher for the SFT than for the FT and SIT tasks.

### Effects of group

**Figure 3:**
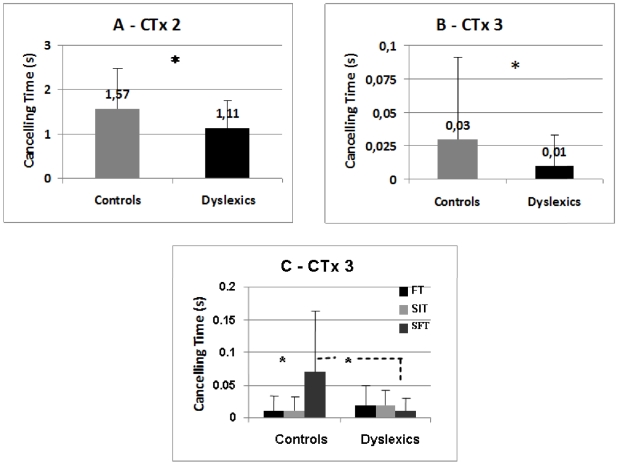
Effects of group in dyslexics vs. controls on the canceling time. This parameter was elaborated from the wavelet transform applied to CoP displacements, of the second (**A** - CTx2) and the third (**B** - CTx3) frequency bands on the mediolateral sway. Asterisk indicates significant difference. Interaction between groups and tasks for the CTx3 (**C**); asterisk indicates significant difference.

There was a main effect of group only for two of the parameters elaborated from the wavelet transform applied to CoP displacements, the canceling time (CT) of the second, medium frequency band (CTx2) and of the third, high frequency band (CTx3) both for the mediolateral sway (respectively F_(1,25)_  = 6.63; p = .012 and F_(1,25)_  = 4.16; p = .045 – see Figure 3a,b). Controls showed longer canceling time for these frequency bands than dyslexics.

### Group task interaction

The only significant interaction between group and task was on the parameter CTx3, i.e. the canceling time for the high frequency band was longer in controls than in dyslexics for the SFT task only (p<0.05).

## Discussion

This study shows that complex Stroop task applied in 15 years old teenagers influences only the variance of speed of body sway and some of the time-frequency parameters; some of these subtle modulations can be different in dyslexics. Next we will discuss these findings.

### Task effect on variance of speed only

First, the interference task had no significant effect on posture stability for any of the parameters when compared with fixation task. This despite the fact that, during the interference task, subjects named loudly the color of the words. Our observations of no effect for 15 years old teenagers contrast those for younger children (7–9 years old) reported by Olivier et al. [8]; indeed a decrement of postural stability was observed in their interference task particularly when vibration over the Achilles tendon and over the insertion of the anterior tibialis was applied. Perhaps the difference is related to the age of subjects. Adolescents in the present study behaved as the adults in the interference Stroop task of the study of Olivier et al. [8] who did not show posture deterioration.

The most important task effects were observed for the flexibility task. To our knowledge this is the first time this task has been used with posturography. The sensitive parameter was the variance of speed. It was significantly higher in the flexibility task relative to the fixation task. Despite the high variability in the former task (see Fig. 2a) individual means were consistently higher thus leading to a statistically significant difference compared to the fixation task. If indeed the effect was produced by variable means alone no statistical significance would be achieved as the statistical test takes into account variability for each task. Thus the flexibility task produces higher variance of speed. As discussed by Kapoula and Lê [31] and Lê and Kapoula [32], this parameter is believed to reflect the energy required for stabilizing the body. Thus increase in variance of speed indicated more energy needed to maintain postural stability while switching from color naming to word reading.

The flexibility task is particularly cognitively demanding, as the subject has to switch strategy for one item to the next. These observations indicated that increase in the difficulty of cognitive task required more energy to maintain postural stability; this is in line with the study of Lacour et al. [7] and consistent with many other studies of elderly or adults, indicating that the effects of cognitive tasks on the postural control could be positive or negative depending on the type of the task used [4], [5], [6]. It remains to be understood by what mechanism the flexibility task influences postural stability more than the interference one. Perhaps it is the change in mental strategy that requires more energy to keep body sway small. Further research which might involve local analysis comparing posture while processing successive items requiring different (i.e. naming color – reading word) versus same processing could be of interest. Here we would argue that the flexibility task is of ecological value as it can probe the capacity to maintain body stability while switching cognitively strategies. In everyday life we have to change cognitive strategies from one instant to the next and in parallel, we do have to maintain postural stability. The flexibility task requires that the two tasks are kept in the working memory; a higher-level process (i.e. additional loop) would allow switching between the two tasks. One could consider the flexibility task not as a double-task but as multiple tasks. Moreover, different cortical/subcortical circuits including frontal and parietal areas are activated for naming color vs. reading the words. Changes in the cortical-subcortical circuitry engaged from one instant to next could be reflected in the energy needed to control posture. Our observations are in line with many other studies [2], [3] reporting that increased contribution of cortical structures affects balance abilities. They are in line with Yardley et al. [33] who reported that interference between a mental task and postural control could be attributed to the attention demands of both tasks. Also, as discussed by Olivier et al. [8], when concurrent tasks can be performed with the available capacity, posture performances could not be affected; adversely, interference could occur when task requirements exceed the capacity of the central nervous system. This would explain the non-effect for the interference task but the effect of the flexibility task.

### Effects of task in the frequency domain – wavelet analysis

As discussed by Lacour et al. [7], the postural instability index (PII) is a relevant physiological parameter giving the information about the postural control in the frequency domain. Indeed, our data show a tendency for the PII to be higher in the flexibility than for the interference or the fixation tasks. Further analysis of the spectral power index indicates mostly effects for the lateral body sway for the medium and high frequency bands. There is a higher spectral power index for the flexibility task than for the interference and fixation tasks for the second, medium frequency band. It is hypothesized that the low frequencies correspond to visual and vestibular control of posture while medium range frequencies are corresponding to cerebellar control [28], [29], [30]. Thus, the higher complexity of the cognitive processes involved in the flexibility task is reflected in the medium ranges of frequencies hypothetically related to cerebellar control. Cerebellar control of posture becomes less efficient as the capacity is shared by the concurrent task with increased cognitive difficulty. Mutually, we observed increased canceling times for the high frequency band in the flexibility task (relative to the other two tasks) indicating a decrease of the fast reflexive loop (e.g. the spinal cord as suggested by Golomer, et al. [34], Kohen-Raz, et al. [29], Paillard, et al. [30]. Taken together these two modulations one could speculate that cognitive complexity translates to less efficient cerebellar control of posture for both dyslexic and non-dyslexic teenagers, and for non-dyslexics only, more efficient control of the reflexive spinal loop controllers. We conclude, in line with Lacour et al. [7], on the usefulness and physiological relevance of the additional parameters provided by the wavelet analysis. The differences between dyslexics and controls will be discussed further below.

### Subtle differences in the time domain between dyslexics and non dyslexics

Here we showed that even though the interference and flexibility tasks were cognitively highly demanding there was no specific deterioration in the basic parameters of posture for dyslexics relative to controls. The wavelet analysis revealed shorter cancellation times for dyslexics for the medium frequency band which is hypothesized to be controlled by the cerebellum; cancellation times were shorter irrespective of the task. Perhaps the cerebellum control of posture is less efficient for dyslexics than non-dyslexics. This observation is compatible with theoretical framework suggesting a cerebellum deficit in dyslexia being responsible of various difficulties with reading, writing and spelling [35], [36]. Nicolson & Fawcett [35] propose a direct impairment of balance and motor skills due to cerebellum impairment in dyslexia. If such were the case, one would expect more dramatic effects on posture (e.g. increase of spectral power and/or increase of postural instability index); the effect we observed is subtle concerning time only, thus suggesting less efficiency than a real deficit. Moreover, the cerebellum is highly involved on eye movement control and fixation stability, e.g. preventing abnormal micro-saccades and attention shifts [37], [38]. Future studies are of interest combining eye movement recording and fixation stability as well as wavelet analysis of posture performances in order to understand better how body control and cognition are interacting. Fixation instability in younger dyslexics has been shown [16]. Attention mechanisms, may again underline the subtle differences revealed in the time frequency domain.

The second subtle difference is specific to the flexibility task. Non dyslexics showed significant increase of the cancellation time of high band frequencies in the most difficult task (the flexibility), while there was no significant effect for dyslexics. Individual inspection of the data showed that 60% of the non-dyslexic teenagers increased this cancellation time versus 40% of the dyslexics; increase times were higher for the former. Increasing the cancellation time of high frequencies somehow improved stability during the flexibility task, while dyslexics maintained the same behavior in all tasks; standard deviation of cancellation times was always small for the group of dyslexic teenagers (see Fig. 3). The few dyslexics who also increased slightly their cancellation time had moderate or severe reading difficulties, and we could not identify specific profile. Again, the difference between dyslexics and non dyslexics is subtle and concerns only time. The interplay between body sway and cognition might be different in dyslexics without leading to clear posture deterioration. We conclude that the strategy of decreasing the time of use of high frequency reflexive loops is more common among non dyslexics, but this needs confirmation with a larger population.

### Controversy on posture abnormalities in dyslexia – this and other studies

Discussion of this aspect concerns the basic posture parameters which are the subject of study in the literature. Our study goes against the idea of primary postural abnormalities at least for advanced in age dyslexic teenagers. It extends this conclusion even when dyslexics are involved in dual tasks or in multiple level complex tasks such as the flexibility task. When considering basic parameters, in such a complex task, their postural stability decreases but similarly to that observed for non-dyslexic teenagers. Their body sway control strategies might be different but their posture stability is still not overall deteriorated.

It should be noted that in the present study no difference was found between the two groups even in the simple fixation condition while a difference was found in our prior study [9]. The difference could be due either to the younger age of subjects studied in the earlier study, and/or to the longer duration of posturography (51.2 sec. vs. 25.6 sec.); as mentioned problems with keeping sustained attention in young dyslexics could influence postural stability.

Thus, postural behavior in quiet upright stance of dyslexics seems to deviate from that of non-dyslexics only for younger children and when prolonged fixation task is required without any other cognitive or active movement [16]. Rochelle et al. [39] also propose that postural instability in dyslexics could be due to their capacity to maintain their attention. When a cognitive task, or just active eye movements [16], are performed, the difference between dyslexics and non-dyslexics in posture performances is no longer significant. Postural control also improves with age [40], [41]. Note that Pozzo et al. [18] compared postural stability over short periods (25.6 sec.) but for younger dyslexic children (11.5±1.8 years) and reported postural instability relative to controls. Perhaps task specific postural instability in dyslexics exists when the postural control system is still immature.

Taking together, the present study with prior studies argues against persisting primary postural syndrome with age in dyslexia. Another important factor to be considered is the site of subjects' recruitment. A bias towards subjects with postural problems may exist if recruited in clinical structures (hospital, clinical services). In the present study all teenagers examined were from school, not from hospital; such recruitment could be more representative of the general population of dyslexics. A recent study by Vieira et al. [42] used a double task (reading of words with different colors); their task was only roughly similar to ours. It was applied on a group of young dyslexics (11.6 year), another group of dyslexics (12.5 years) wearing prisms and proprioceptive soles for 3 months, and a control group (10.6 years). First, the authors reported no difference between groups in postural stability in the fixation control task. In contrast, for the double task – reading, postural instability was higher for dyslexics than controls; moreover prismatic treatment and soles resolved such difference. Here again the effects are task specific (no effect in the fixation task alone). The decreased posture stability for dyslexics when tested with the double task prior to prism-sole treatment contrasts the absence of the effects in our study. Yet the difference could be explained by several factors: age, the type of double task, a possible recruitment bias as mentioned above – clinic vs. school.

Taken all these considerations, particularly the highly task specific postural effects reported in dyslexics, the most parsimonious explanation would be that postural problems might exist in some young children with dyslexia, perhaps related to development but do not seem typical characteristic of dyslexia. Characterization of such sub-group remains to be done. Based on our past and present study with young and older dyslexics recruited from the school, we conclude that postural instability may appear in young dyslexics only when sustained fixation and attention with no cognitive task are required.
